# Multiple modes of selection can influence the role of phenotypic plasticity in species' invasions: Evidence from a manipulative field experiment

**DOI:** 10.1002/ece3.7311

**Published:** 2021-03-09

**Authors:** Elizabeth P. Lacey, Freddy O. Herrera, Scott J. Richter

**Affiliations:** ^1^ Department of Biology University of North Carolina Greensboro NC USA; ^2^ Department of Mathematics & Statistics University of North Carolina Greensboro NC USA

**Keywords:** correlational selection, costs and benefits, fitness, genetic correlation, invasion, *Plantago lanceolata*, plasticity

## Abstract

In exploring the roles of phenotypic plasticity in the establishment and early evolution of invading species, little empirical attention has been given to the importance of correlational selection acting upon suites of functionally related plastic traits in nature. We illustrate how this lack of attention has limited our ability to evaluate plasticity's role during invasion and also, the costs and benefits of plasticity. We addressed these issues by transplanting clones of European‐derived *Plantago lanceolata* L. genotypes into two temporally variable habitats in the species' introduced range in North America. Phenotypic selection analyses were performed for each habitat to estimate linear, quadratic, and correlational selection on phenotypic trait values and plasticities in the reproductive traits: flowering onset and spike and scape lengths. Also, we measured pairwise genetic correlations for our “colonists.” Results showed that (a) correlational selection acted on trait plasticity after transplantation, (b) selection favored certain combinations of genetically correlated and uncorrelated trait values and plasticities, and (c) using signed, instead of absolute, values of plasticity in analyses facilitated the detection of correlational selection on trait value‐plasticity combinations and their adaptive value. Based on our results, we urge future studies on species invasions to (a) measure correlational selection and (b) retain signed values of plasticity in order to better discriminate between adaptive and maladaptive plasticity.

## Graphical abstract

In nature, we experimentally tested for correlational selection acting on a suite of functionally related trait values and trait plasticities in an invasive species. Our results showed that correlational selection favored certain combinations of genetically correlated and uncorrelated trait values and trait plasticities. Using signed, instead of absolute, values of plasticity in analyses facilitated the detection of correlational selection on trait value–plasticity combinations and their adaptive value.

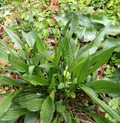

## 1 INTRODUCTION

The role of phenotypic plasticity in species invasions has long been debated (e.g., Baker, 1965; Ghalambor et al., 2007; Richards et al., 2006). Empirical comparisons of ancestral and derived populations, invasive and native noninvasive species, and species varying in invasiveness have provided valuable insights about this role and the evolution of plasticity (e.g., Bossdorf et al., 2005; Colautti et al., 2017; Colautti & Lau, 2015; Davidson et al., 2011; Godoy et al., 2011; Matzek, 2012; Molina‐Montenegro et al., 2012; Morris et al., 2014; Muth & Pigliucci, 2007; Parsons & Robinson, 2006; van Kleunen et al., 2011; Yeh & Price, 2004). However, the debate continues because of the paucity of empirical attention given to several aspects of the invasion process. First, multiple traits, as opposed to a single trait, generally influence the fitness of an individual in a natural habitat. Whereas many empirical studies have examined the role of a single trait value or plasticity on invasion success, few have looked at the role of suites of traits or the responses of selection for genotypes varying in plasticity in nature (Ghalambor et al., 2007, 2015; Hendry, 2016; Lacey et al., 2012). Because of this, the possibility of correlational selection has been ignored. Second, individuals entering a new habitat are likely characterized by genetic correlations among trait values and plasticities, and these correlations could affect the responses to selection (Auld et al., 2010). However, seldom have the interactions between these correlations and modes of selection been examined empirically (Schrieber et al., 2017). Third, costs and benefits of plasticity have generally been assessed using absolute values of plasticity in empirical studies (e.g., van Kleunen & Fischer, 2005, 2007; Palacio‐Lopez et al., 2015; Volis, 2009; Wang et al., 2018). This practice can, however, obscure fitness effects, that is, adaptive versus maladaptive plasticity (Dechaine et al., 2007; van Kleunen & Fischer, 2005, 2007; Weinig et al.. 2006). Below we explore these aspects of colonization, using data from a manipulative experiment in which we transplanted individuals from a species' native range into its introduced range.

When a colonizing individual enters a new habitat, the individual brings a suite of genes that underlie the phenotypic plasticities of multiple traits, that is, underlie the reaction norms often used to describe plasticity. We define plasticity as the ability of a genotype to modify the phenotype of a trait in response to environmental change. Multiple molecular studies now support the existence of such plasticity genes (e.g., Cho et al., 2017; Corl et al., 2018; Des Marais et al., 2013; Gu et al., 2019; Han et al., 2019; Knies et al., 2009; Marshall et al., 2020; Morris et al., 2014; Nilson & Assmann, 2010). Ideally, it would be helpful to have measures of these plasticities, that is, the potential for environmental flexibility in arriving colonists, and have measures of their genetic correlations, for example, among plasticities and trait values. These correlations could potentially influence the range of phenotypes expressed by colonists in a new habitat, thereby affecting the direction and magnitude of early evolution (Antonovics, 1976).

One would also like to identify which plasticities are the targets of phenotypic selection and quantify the direct and indirect effects on fitness (Antonovics, 1976; Endler, 1986; Lande & Arnold, 1983; Mitchell‐Olds & Shaw, 1987). Empirically identifying these targets has not been easy. While we recognize that the phenotypic value of a trait can directly or indirectly affect fitness in a local environment (Via, 1993; Via et al., 1995), the plasticity of that trait may affect fitness directly or indirectly, and independently of the fitness effect of the trait's phenotypic value (Bradshaw, 1965; de Witt & Scheiner, 2004; Mousseau & Fox, 1998; Roff, 1997; Scheiner, 1993; Scheiner & Callahan, 1999; Schlichting, 1986; Sultan, 1987; van Tienderen, 1991; West‐Eberhard, 2003). However, when a phenotypic value of a trait and its plasticity are genetically correlated, estimating independent fitness effects can be more difficult (Auld et al., 2010).

Multiple studies have experimentally demonstrated an adaptive benefit of phenotypic plasticity for many individual traits in natural habitats (e.g., environments varying in: light quality: Dudley & Schmitt, 1996; Galloway & Etterson, 2007; Huber et al., 2004; Schmitt et al., 1995; neighborhood density: Donohue et al., 2001; temperature: Kingsolver & Huey, 1998; Lacey et al., 2012; and inducible defenses: Agrawal, 1998; Relyea, 2004), and with little to no fitness cost (Auld et al., 2010; Van Buskirk & Steiner, 2009; van Kleunen & Fischer, 2005, 2007). Few studies, however, have tested if trait plasticity, independently of the trait value, is a target of selection, and of these, most have considered only directional selection on plasticity (exceptions: Baythavong & Stanton, 2010; Callahan & Pigliucci, 2002; Donohue et al., 2000; McIntyre & Strauss, 2014; Scheiner & Berrigan, 1998; Steinger et al., 2003; Tucić et al., 1998). Similarly, few empirical studies of colonists have quantified the independent and correlated effects of a trait's value and its plasticity on fitness (Colautti & Lau, 2015; Davidson et al., 2011; Godoy et al., 2011; Matzek, 2012). Ideally, we would like estimates of selection on plasticity that account for both the independent effects of trait phenotypic values and plasticities and the genetic correlations among phenotypic values and plasticities for these functionally related traits (e.g., Endler, 1986; Kingsolver et al., 2001; Lande & Arnold, 1983).

We would also like estimates of linear and nonlinear modes of selection (Antonovics, 1976; Endler, 1986; Lande & Arnold, 1983; Mitchell‐Olds & Shaw, 1987). Theoretically, selection can be linear (directional), quadratic (e.g., stabilizing, disruptive), or correlational, and selection on various combinations could potentially contribute to the evolution of a trait value and its plasticity in natural populations. Quadratic and correlational selection are expected to be common in nature (Arnold et al., 2008; Blows & Brooks, 2003; Roff & Fairbairn, 2007; Schluter & Nychka, 1994; Sinervo & Svensson, 2002), and in particular, correlational selection, that is, selection that favors certain combinations of traits, is expected to cause the evolution of genetic correlations. Measures of this nonlinear mode on plasticity in nature are very rare (Donohue et al., 2000; Roff & Fairbairn, 2012; Tucić et al., 1998), as they are for trait values generally (e.g., Kingsolver et al., 2001, 2012).

In thinking about the possible roles of plasticity in the process of early colonization and evolution in a new landscape, we envision that plasticity could influence the process in several ways. (a) Plasticity could, by itself, be adaptive, or maladaptive, thereby directly causing the mean fitness of a population to move toward or away from a fitness peak, respectively, as defined by the new adaptive landscape (e.g., Ghalambor et al., 2007; Hendry, 2016). (b) Plasticity could indirectly facilitate or impede movement toward a fitness peak because of a pre‐existing genetic correlation with a correlated trait value or plasticity that is, itself, under direct selection (Auld et al., 2010; Ghalambor et al., 2007). (c) Plasticity could facilitate or impede movement toward multiple fitness peaks because of correlational selection, which favors multiple combinations of traits involving plasticity. (d) Plasticity could facilitate the maintenance of genetic diversity when a fitness peak is broad.

To explore these possibilities, we conducted an experiment that assessed the fitness effects of trait value and plasticity in reproductive traits in two temporally variable habitats in nature. Our habitats were far enough apart that a population in one habitat would not experience the environment of the other. Habitats resembled each other in terms of photoperiod but differed in temperature regime (Figure S1). Also, each habitat was itself temporally variable (e.g., with respect to temperature, precipitation). Temporal variability could favor the evolution of plasticity, depending on the frequency and pattern of variation (Gabriel & Lynch, 1992; Gomulkiewicz & Kirkpatrick, 1992; Marshall et al., 2019; Moran, 1992; van Tienderen, 1991) We focused on reproductive traits of *Plantago lanceolata* L. because they contribute strongly to reproductive success, and, thus, to individual fitness and because the thermal environment during the long reproductive season is predictably variable at scales of weeks and months, as well as being diurnally variable.

Clones of native European *Plantago lanceolata* genotypes were transplanted into two habitats within the species' introduced range in North America. To expand the range of phenotypic variation upon which selection could act, we used genotypes derived from populations that spanned most of the latitudinal range of the species in Europe. Expanding phenotypic variation beyond that which currently exists in a habitat increases the probability of detecting selection (cf. Mitchell‐Olds & Shaw, 1987; Wade & Kalisz, 1990). Our study allowed us to explore how different modes of selection on plasticity might have affected the early evolution of *P. lanceolata*, given initial trait correlations. Our first goal was to determine which reproductive traits showed genetic variation in plasticity. We defined plasticity as a property of a genotype and as the ability, or flexibility, to modify its phenotype in response to environmental change. Focusing on this subset of traits, we then addressed the following questions for our European genotypes at each transplant site: (a) What are the genetic correlations between trait values and plasticities? (b) What is the most appropriate statistical model (e.g., linear, quadratic, correlational) for assessing selection on plasticities and trait values, given our data? (c) What modes of selection are acting on trait values and plasticities? Here we looked for evidence of directional, quadratic (i.e., curvilinear), and correlational selection. (d) How are genetic correlations expected to affect the responses to selection? (e) What are the effects of using signed values of plasticity versus using absolute values to estimate costs and benefits of plasticity? Using clones of the same European genotypes provided the statistical power for these assessments.

## 2 METHODS

### 2.1 The study species and focal traits

*Plantago lanceolata* L. (English, or ribwort, plantain), Plantaginaceae, is a temperate, weedy, gynodioeceous, herbaceous perennial, native to Eurasia but now well established in disturbed areas, lawns, and grasslands in North America (Cavers et al., 1980). Many traits show intragenerational and intergenerational plasticity (Bradshaw, 1965; Caseet al., 1996; Lacey & Herr, 2000, 2005; Primack & Antonovics, 1981; Schmitt et al., 1992; van Hinsberg, 1997; van Tienderen, 1990, 1992; van Tienderen & van der Toorn, 1991a, 1991b; Wolff & van Delden, 1987). Throughout the reproductive season, which in some regions lasts for 6 months, spikes (inflorescences) of tightly packed flowers on leafless scapes rise from basal rosettes. One can usually see multiple spikes in different stages of development on a plant once reproduction begins. Spike development is sequential and overlapping. Floral development on a single spike and scape elongation on that spike are influenced by temperature and can continue for several weeks (Lacey, pers. Obs.). The time from spike appearance to seed maturity on the spike typically ranges from 2 to 6 weeks, depending on the temperature and water availability. Floral reflectance is temperature‐sensitive, and consequently, spikes of flowers change color throughout a reproductive season (Lacey & Herr, 2005; Stiles et al., 2007). Protogynous flowers are self‐incompatible (Ross, 1973; van Damme, 1983) and predominately wind‐pollinated (Cavers et al., 1980). Flowering onset and end times are predominately photoperiodically controlled (Snyder, 1948).

We initially looked at six reproductive characters: flowering onset, duration of flowering season, median flowering time, spike length, spike number, and scape length. All can potentially contribute to an individual's fitness. Onset and duration are generally under selection to coincide with the arrival and cessation, respectively, of environmental conditions suitable for reproductive success. Median flowering time, and spike number and length are indicators of how a plant allocates its resources to reproduction within a reproductive season. Reproductive allocation in a perennial species is subject to natural selection because of its potential to influence future survival. Median flowering time describes the temporal allocation pattern within a reproductive season. Spike number and length describe the spatial pattern of allocation within a plant. Both patterns are potentially subject to selection by seed predators and pathogens and their time of appearance (e.g., Lacey et al., 2003). Finally, scape length in a wind‐pollinated species should influence pollen dispersal distance and possibly also the temperature of reproductive tissues. Longer scapes should increase the exposure of reproductive tissues to cooling by wind, whereas shorter scapes should enhance radiative warming from the ground.

### 2.2 Genotype selection

In 2010, 50 European genotypes from 14 populations (1–4 genotypes/population) used in an earlier study (Lacey et al., 2010) were chosen from the latitudinal range of the species in Europe (Latitude = 41–62°N: Italy to Scandinavia) and across the range of thermal plasticities in floral reflectance (Lacey et al., 2010). This variation was expected to expand the range of phenotypic plasticity for the focal reproductive traits. All populations grew at altitudes <250 m above sea level. Parents of the experimental genotypes had been collected as seeds in Europe in 2000. To reduce maternal effects, parents had been grown in a greenhouse and then isolated by population for wind pollination and seed production under similar environmental conditions (Lacey et al., 2010).

### 2.3 Transplant sites

Two 3 × 3 m transplant plots were established in open fields, one at The North Carolina Agricultural and Technical State University Farm (NCAT) in Greensboro, NC, USA (36°06′N, 79°73′W: altitude = 272 m), and one at the Mountain Lake Biological Station (MLBS) on top of Salt Pond Mountain, VA, USA (37°37′N, 80°52′W; altitude = 1,181 m). We chose these sites because they are similar in photoperiod (2015 data: 14 hr daylength reached on May 11 and May 13 at Blacksburg, VA. and Greensboro, NC, respectively), but differ in the thermal environment during the growing season. MLBS has, on average, a colder and shorter reproductive season with a greater range of temperatures (Figure S1: maximum temperature – minimum temp. from April 1 to October 1: MLBS = 23.8°C; NCAT = 19.5°C). A few scattered *P. lanceolata* growing naturally with forbs and grasses at both sites were removed from the plots before transplanting. During the experiment, we trimmed the vegetation surrounding transplants so that we could find *P. lanceolata* spikes.

### 2.4 Experimental design

We grew clones of the experimental genotypes in teacups (approx. 473 ml) under short days in four growth chambers to promote vegetative growth (2 months, 20°C, 10 hr day/15°C, 14 hr night; daily watering, 1/2–strength Hoagland's 3X/week, no more than 2 clones/genotype per chamber). Then in 2011, we transplanted three clones of similar rosette size for each of the 50 genotypes (except 2 clones for 2 genotypes at MLBS) into each plot. MLBS clones were made and transplanted 6 weeks later than NCAT clones so that MLBS clones would resemble NCAT clones in size and so that the MLBS environment at transplant time would better resemble the NCAT environment at transplant time (NCAT: March 4, Julian Day 86; MLBS: April 15, Julian Day 105). MLBS not uncommonly has snow‐covered or frozen ground in early March. Clones were randomly assigned positions 20 cm apart. That summer, we recorded and marked biweekly spike production per clone. As spikes matured, we collected the spikes and recorded scape and spike lengths.

For our analyses, flowering onset of each clone was measured as the week of first spike appearance based on Julian days. Flowering duration was measured as the number of weeks between first and last spike appearance. Median week of spike production served as the proxy for the timing of spike production within the flowering season. Median week was the number of weeks that a clone took to reach 50% total spike production, starting from flowering onset. Because each clone produced multiple spikes and scapes, we calculated the mean length for each of these two traits for up to the first 20 scapes/spikes produced per clone and used the mean values per clone as the trait values for spike and scape length in the selection analyses. Scape lengths within an individual clone varied little over time (Figure S2). Most plants stopped flowering by the 20th spike (Herrera, 2013). Spike lengths predictably declined throughout the reproductive season, which is typical of declining resources allocated to reproduction as a reproduction season progresses (Figure S3 and Lacey et al., 2003).

Total seasonal seed production, the fitness proxy for each clone, was estimated by first estimating seed production per spike. We individually weighed a sample of spikes and counted their seeds (spike sample size: MLBS = 53, NCAT = 69). Linear regression models (PROC REG, SAS version 9.4, 2008) of these data were used to estimate seed production for all other spikes (MLBS: seeds = 380.6891 [spike weight], *r* ^2^ = .82; NCAT: seeds = 403.4024 [spike weight], *r* ^2^ = .77). Seed numbers were summed over spikes for each clone.

### 2.5 Plasticity measurements

By necessity, plasticity, a genotype's ability to modify a trait in response to environmental change, was measured at the genotype level rather than the clonal level. Plasticity for each reproductive trait per genotype was determined by subtracting the mean value of a genotype's trait (i.e., mean value of clones per genotype) at MLBS from its mean value at NCAT. Thus, we examined the effects of both magnitude and direction of plasticity as expressed in natural settings. A positive value meant that a trait had a higher mean trait value at NCAT, whereas a negative value meant that a trait had higher value at MLBS. This method provided information about the direction of phenotypic change, which would be lost if we used absolute values of plasticity in analyses. In the Discussion, we address the effects of using signed versus absolute values of plasticity. Calculating plasticity as the difference between trait means per site raised the question of whether or not a trait's value and plasticity were correlated with each other. Our analyses addressed this possibility.

### 2.6 Statistical analyses

For statistical analyses, we used the 34 genotypes that flowered at both sites. More clones per genotype flowered at NCAT than at MLBS, and the number of clones differed among genotypes. At NCAT, all clones (3) flowered for each of 17 genotypes, 2 flowered for 15 genotypes, and 1 flowered for 2 genotypes. At MLBS, 3 clones flowered for 9 genotypes, 2 flowered for 11 genotypes, and 1 clone flowered for 14 genotypes. This yielded a total of 146 clones representing 34 maternal families and 14 European populations for our statistical analyses.

First, using PROC MIXED (SAS version 9.4), we examined the effect of transplant site and genotype separately on six reproductive traits: flowering onset, flowering duration, median week of spike production, total seasonal spike number, scape length, and spike length. G (Genotype) × E (Site) interactions were used to identify which traits were genetically variable for plasticity. Subsequent analyses were restricted to traits for which interactions were statistically significant (*p* < .05). These traits, in contrast to those showing only significant E(Site) effects, showed evidence of genetically different responses to the experimental sites, that is, showed evidence that the sites favored some genotypes over others based on their plasticity. We did not include genetically variable traits lacking plasticity, so that the number of observations in our dataset would exceed the number of predictors in selection models. Before all analyses, trait values and plasticities were standardized to a mean = 0 and variance = 1 within site.

To see if local microsite variation affected the covariances between trait means and fitness, we used multilevel regression models (PROC MIXED) to conduct both phenotypic and genotypic selection analyses on the trait values. Local microsite variation can produce biased phenotypic results if the microsite variation alters the covariances (Lande & Arnold, 1983; Rausher, 1992). Such covariances can result either from environmental conditions that independently influence fitness and the focal traits or from selection on unmeasured traits that are themselves correlated with the traits of interest. Results of the regression analyses showed that the differences between genotypic and phenotypic trends were negligible, that is, there was little evidence of microenvironmental bias (Figure S4). Therefore, we conducted all selection analyses using phenotypic measures for our trait values. This took advantage of all our data and increased our sample size and statistical power to detect selection.

Phenotypic selection (multivariate regression) analysis was used to estimate the direction and intensity of linear (directional) and nonlinear (quadratic and correlational) selection on each trait found to be genetically variable for plasticity (Lande & Arnold, 1983; Phillips & Arnold, 1989; van Tienderen, 1991). We used multilevel regression models (PROC MIXED) instead of least squares regression because clonal data were grouped by genotype. Multilevel regression properly incorporates predictors associated with both clone and genotype. This allowed us to calculate error terms at the clonal level for trait values and error terms at the genotypic level for plasticity (e.g., Raudenbush & Bryk, 2002; Singer, 1998). Also, we could examine the effects of plasticity predictors on our fitness proxy, independent of any possible correlations between trait values and trait plasticities. The number of observations in our models exceeded the number of independent predictors.

In order to determine the most efficient model for analyzing our data, we compared various subset multivariate regression models per site, depending on the question being addressed. The form for the full model 1 (example shown here includes 2 of the 3 traits) was:

$$\begin{array}{cc} W & =\alpha +\beta_{i}X_{i}+\gamma_{\mathrm{ii}}X_{i}^{2}+\beta_{\mathrm{pi}}pX_{i}+\gamma_{\mathrm{pipi}}{\left(pX_{i}\right)}^{2}+\gamma_{\mathrm{ipi}}X_{i}\times pX_{i}\\ & \;+\beta_{j}X_{j}+\gamma_{\mathrm{jj}}X_{j}^{2}+\beta_{\mathrm{pj}}pX_{j}+\gamma_{\mathrm{pjpj}}{\left(pX_{j}\right)}^{2}+\gamma_{\mathrm{jpj}}X_{j}\times pX_{j}\\ & \;+\gamma_{\mathrm{ij}}X_{i}\times X_{j}+\gamma_{\mathrm{ipj}}X_{i}\times pX_{j}+\gamma_{\mathrm{pij}}pX_{i}\times X_{j}+\gamma_{\mathrm{pipj}}pX_{i}\times pX_{j}+latitude+\varepsilon +\varepsilon_{g} \end{array}$$

where *W* is the fitness proxy at a transplant site, *α* is a constant, *X_i‐j_* are the site trait values, and *pX_i‐j_* are the plasticity values of traits. Because traits were measured on clones nested in genotype, a multilevel model with random intercepts was fit, and thus, the model included error terms for both clone (*ε*) and genotype (*ε*_g_). Linear selection gradients (e.g., *β_i_*, *β_j_*) estimated the strength of directional selection on trait values, plasticities, and latitude. Linear selection gradients were estimated from models that included only linear terms. Quadratic selection gradients (e.g., *ɣ_ii_*, *ɣ_jj_*) measured the curvature in fitness functions and also estimated stabilizing (e.g., negative *ɣ_ii_*) and disruptive (e.g., positive *ɣ_ii_*) selection on a trait value or plasticity. Cross‐product selection gradients (e.g., *ɣ_ij_*, *ɣ_i_*_p_ *_i_*, *ɣ_j_*_p_ *_j_*, *ɣ_i_*_p_ *_j_*, *ɣ*_p_ *_ij_*, *ɣ*_p_ *_i_*_p_ *_j_*) estimated correlational selection between pairs of trait values and trait plasticities. This type of model measures the change in distribution of a trait value or plasticity due to selection acting directly on the predictor, independent of changes due to correlations with other predictors included in the model (Lande & Arnold, 1983; Mitchell‐Olds & Shaw, 1987). A positive *ɣ_ij_* value indicated that natural selection favored a positive correlation, whereas a negative *ɣ_ij_* indicated that selection favored a negative correlation. Because the experimental genotypes were progeny of parents collected from a range of latitudes in Europe, we included a linear term to account for source latitude of the parents. Quadratic coefficients and their standard errors were doubled (Stinchcombe et al., 2008).

To address the question of whether or not plasticity was a target of selection at each site, we compared the full model with a model including all trait values but lacking all plasticity predictors. Second, to address whether linear, quadratic, and/or correlational selection had occurred, we compared the full model with the following reduced models: (a) a “quadratic” model with only linear and quadratic terms, (b) a “cross‐product” model with only linear and cross‐product terms, and (c) a “linear” model with only linear terms. The goal was to determine if we could reduce the full model at each site and not lose predictive power to estimate fitness. We felt that this was the best way to address the global question of whether there was statistical evidence for a particular type of selection (e.g., quadratic). For example, our alternative hypothesis for the quadratic selection test was that there was at least one nonzero coefficient among the quadratic selection terms. If we did not have evidence to support this, we proceeded to drop the quadratic terms and refit the model. We compared the log‐likelihood statistics of each pair of models using a chi‐square test to determine if the full model was a better fit, or if we could reduce the full model by eliminating a set of predictors without losing much predictive power. Finally, using the most efficient model for each site, we examined the selection coefficients of individual trait values and plasticities.

The selection coefficients can be used to construct selection surfaces (Lande & Arnold, 1983; Phillips & Arnold, 1989). Therefore, to visualize how correlational selection might act on pairs of trait values and/or plasticities, we used PROC TPSPLINE (SAS version 9.4) to produce fitness surfaces for the pairs. This nonparametric procedure can complement the selection gradient analyses by revealing subtleties in selection surfaces (Brodie et al., 1995; Schluter & Nychka, 1994).

Finally, we calculated pairwise genetic correlations between trait values, plasticities, and source latitude for our traits at the genotypic level **(**PROC CORR) so that we could consider these correlations when interpreting our selection results. For each trait value, clonal values were averaged within genotype and site, and the means were used to estimate pairwise correlations within sites. Unlike genetic correlations calculated from a breeding design, our correlation values included contributions from additive, dominance, and epistatic effects. Maternal effects had been reduced because the experimental genotypes were the progeny of parents that had been grown and crossed in similar environments (See Lacey et al., 2010). Also, the experimental genotypes had been grown together in a greenhouse for several years before conducting this experiment.

## 3 RESULTS

**FIGURE 1:**
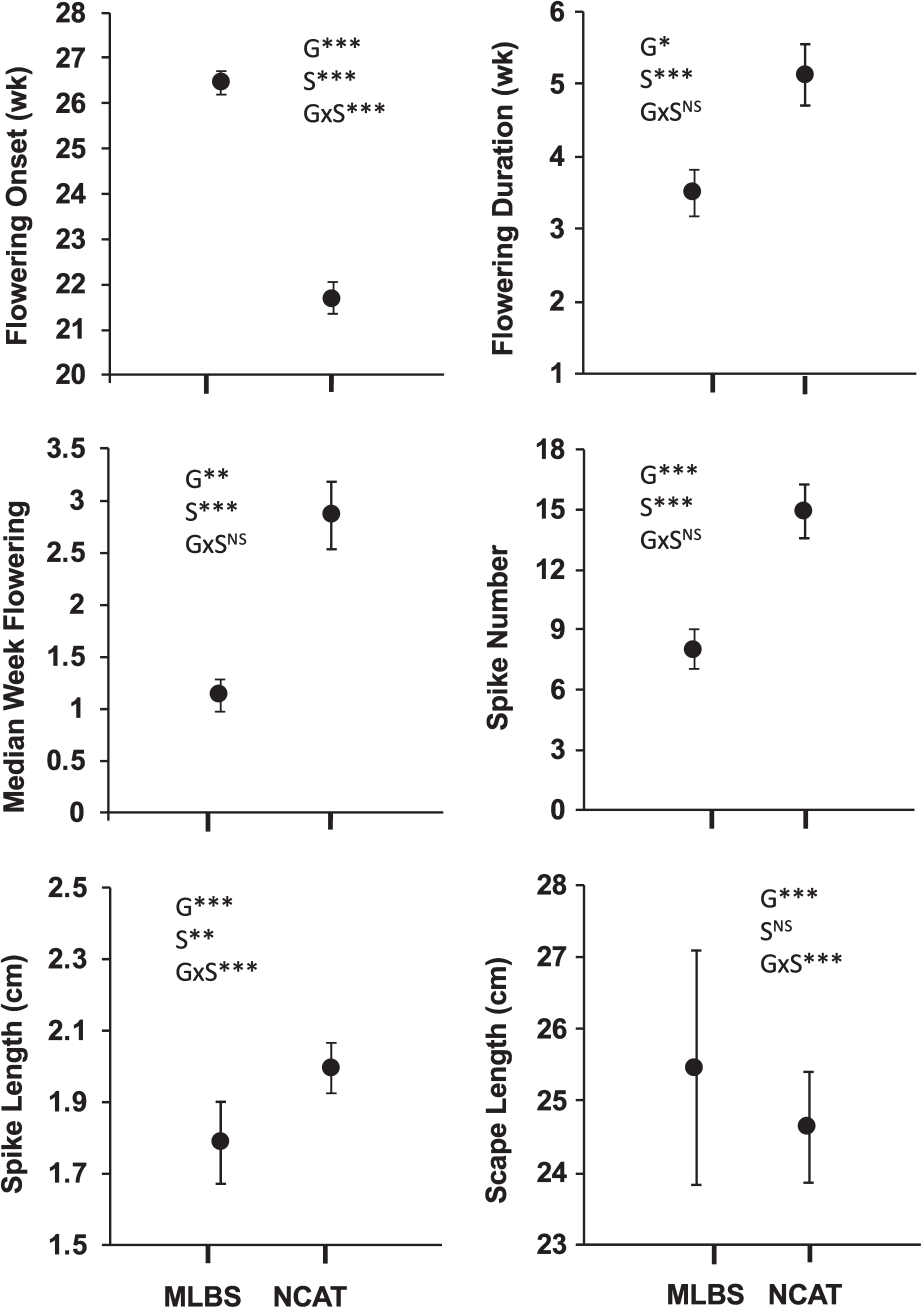
Phenotypic trait means ± 1 *SE* at MLBS (cooler) and NCAT (warmer) transplant sites. Significance levels (**p <.01, ***p <.001, NS = *p* > .05) are shown for effects of genotype (G), transplant site (S), and genotype by site interaction (G × S)

Flowering began earlier and lasted longer at NCAT than at MLBS, as evidenced by genotypic differences in flowering onset, median week of flowering, and duration (Figure 1 and Figure S1: NCAT week 17–31 [April–August] versus MLBS week 25–34 [June–September]). Also, genotypes at NCAT produced more and longer spikes (Figure 1). Site differences were statistically significant for all traits except scape length. All traits showed genetic variation, but only flowering onset, scape length, and spike length showed evidence of genotypic variation in plasticity, that is, genotype by site interactions. Therefore, the following analyses addressed these three traits.

Pairwise genetic correlations differed between sites (Table 1). At MLBS, all genetic correlations between spike and scape lengths and plasticities were large and highly significant. Scape and spike lengths were positively correlated with each other, as were scape and spike length plasticities. In other words, genotypes with longer spikes had longer scapes and showed less plasticity, that is, less variation, in these traits. At NCAT, flowering onset and plasticity were strongly positively genetically correlated, and both were strongly negatively correlated with spike length. In other words, later flowering genotypes produced shorter spikes and showed greater plasticity in flowering onset. Plasticities in spike and scape lengths were strongly positively correlated; genotypes more plastic for spike length were also more plastic for scape length. Other pairwise genetic correlations were small, and no trait was strongly correlated with latitude at either site.

**TABLE 1:** Genetic Pearson correlation coefficients (above) and *p* values (below) between independent variables used in phenotypic selection analyses

MLBS	Onset trait value	Onset plasticity	Spike length trait value	Spike length plasticity	Scape length trait value	Scape length plasticity	Source latitude
NCAT							
Onset trait value		−0.31156 0.0729	0.35583 0.0389	0.08616 0.628	0.08976 0.6137	−0.29226 0.0935	−0.17278 0.3285
Onset plasticity	0.71397 <0.0001		−0.18797 0.2871	−0.23515 0.1807	0.06787 0.7029	−0.10027 0.5726	0.25573 0.1444
Spike length trait value	−0.47585 0.0044	−0.60944 0.0001		−0.64251 <0.0001	0.69742 <0.0001	−0.88814 <0.0001	−0.23735 0.1765
Spike length plasticity	−0.15839 0.371	−0.23515 0.1807	0.30964 0.0747		−0.81557 <0.0001	0.78917 <0.0001	0.25414 0.1469
Scape length trait value	−0.05214 0.7696	−0.28125 0.1071	0.35426 0.0398	0.33943 0.0495		−0.74457 <0.0001	−0.15379 0.3852
Scape length plasticity	−0.30996 0.0744	−0.10027 0.5726	0.23592 0.1792	0.78917 <0.0001	0.10378 0.5592		0.15087 0.3944
Source latitude	0.11398 0.521	0.25573 0.1444	−0.18297 0.3003	0.25414 0.1469	0.1732 0.3273	0.15087 0.3944	

Note

MLBS data are in the upper right, and NCAT data are in the lower left.

Patterns of Selection at *MLBS:*

A comparison of the full multilevel regression model with that lacking plasticity predictors showed that all plasticity predictors could be eliminated from the full model without loss of predictive power at MLBS. The chi‐square test indicated that the two models did not significantly differ from each other (*Χ* ^2^ = 14.1, *p* = .722, *df* = 18: full model: −2 log‐likelihood = 102.5, no‐plasticity model: −2 log‐likelihood = 116.6). Thus, there was little evidence of plasticity being a target of selection at MLBS. A comparison of the full model (1) with reduced models (2–4) showed that the full model predicted fitness significantly better than any reduced model (log‐likelihood difference from linear model = 144.7, *Χ* ^2^ = 42.2, *p* = .0040, *df* = 21; difference from linear + cross‐product model = 125.2, *Χ* ^2^ = 22.7 *p* = .0009, *df* = 6; difference from quadratic model = 141.1, *Χ* ^2^ = 38.6 *p* = .0007, *df* = 15). Based on these results, we used the full model lacking plasticity predictors, but including linear, quadratic, and cross‐product predictors, to examine nonlinear selection on individual traits (Table 2A).

**TABLE 2:** Linear and nonlinear selection coefficients (±1 *SE*) and *p* values from phenotypic selection analyses of effects of trait values, plasticities (plast) and cross‐products on fitness at each transplant site

Variable	Linear (*β*)	*p*	Quadratic (2*ɣ_ii_*)	*p*	Cross‐product (*ɣ_ij_*)	*p*
MLBS						
Onset time	**−0.8475 ± 0.2311**	**.0011**	**−1.8492 ± 0.6496**	**.0100**		
Spike length	**0.6222 ± 0.1356**	**<.0001**	**0.1106 ± 0.1738**	**.0006**		
Scape length	−0.0310 ± 0.1954	.8753	0.2584 ± 0.1742	.1535		
Onset time*spike length	**‐**	**‐**	**‐**	**‐**	**−0.5098 ± 0.1805**	**.0105**
Onset time*scape length	**‐**	**‐**	**‐**	**‐**	**0.4159 ± 0.1669**	**.0216**
Spike length*scape length	**‐**	**‐**	**‐**	**‐**	**−0.4908 ± 0.1216**	**.0006**
Latitude	−0.0373 ± 0.0259	.1607	‐	‐		
NCAT						
Onset time	−0.2016 ± 0.1655	.2294	‐	‐		
Onset plasticity	0.0391 ± 0.1282	.7627	‐	‐		
Spike length	**0.2408 ± 0.1078**	**.0304**	‐	‐		
Spike plasticity	−0.2385 ± 0.1766	.1873	‐	‐		
Scape length	−0.0110 ± 0.1192	.9268	‐	‐		
Scape plasticity	0.0951 ± 0.1755	.5922	‐	‐		
Onset time*onset plast	‐	‐	‐	‐	−0.1238 ± 0.1399	.3579
Onset time*spike length	‐	‐	‐	‐	0.1432 ± 0.2062	.4912
Onset time*spike plast	‐	‐	‐	‐	0.0801 ± 0.2593	.7594
Onset time*scape length	*‐*	*‐*	*‐*	*‐*	*−0.5409 ± 0.2731*	*.0558*
Onset time*scape plast	‐	‐	‐	‐	−0.2368 ± 0.2405	.3318
Onset plast*spike length	**‐**	**‐**	**‐**	**‐**	**−0.3854 ± 0.1704**	**.0302**
Onset plast*spike plast	‐	‐	‐	‐	0.2361 ± 0.1818	.2054
Onset plast*scape length	**‐**	**‐**	**‐**	**‐**	**0.4025 ± 0.1962**	**.0480**
Onset plast*scape plast	‐	‐	‐	‐	−0.2021 ± 0.1795	.2706
Spike length*spike plast	**‐**	**‐**	**‐**	**‐**	**−0.6410 ± 0.1972**	**.0026**
Spike length*scape length	*‐*	*‐*	*‐*	*‐*	*−0.2095 ± 0.1062*	*.0568*
Spike length*scape plast	**‐**	**‐**	**‐**	**‐**	**0.4442 ± 0.1770**	**.0170**
Spike plast*scape length	**‐**	**‐**	**‐**	**‐**	**0.8261 ± 0.2897**	**.0074**
Spike plast*scape plast	**‐**	**‐**	**‐**	**‐**	**0.1713 ± 0.0774**	**.0358**
Scape length*scape plast	**‐**	**‐**	**‐**	**‐**	**−1.0602 ± 0.2525**	**.0002**
Latitude	**−0.0461 ± 0.0189**	**.0211**				

Note

Traits = flowering onset time, spike length, and scape length. All quadratic values are doubled. Linear coefficients are from the linear model. Boldfaced = *p* < .05.

**FIGURE 2:**
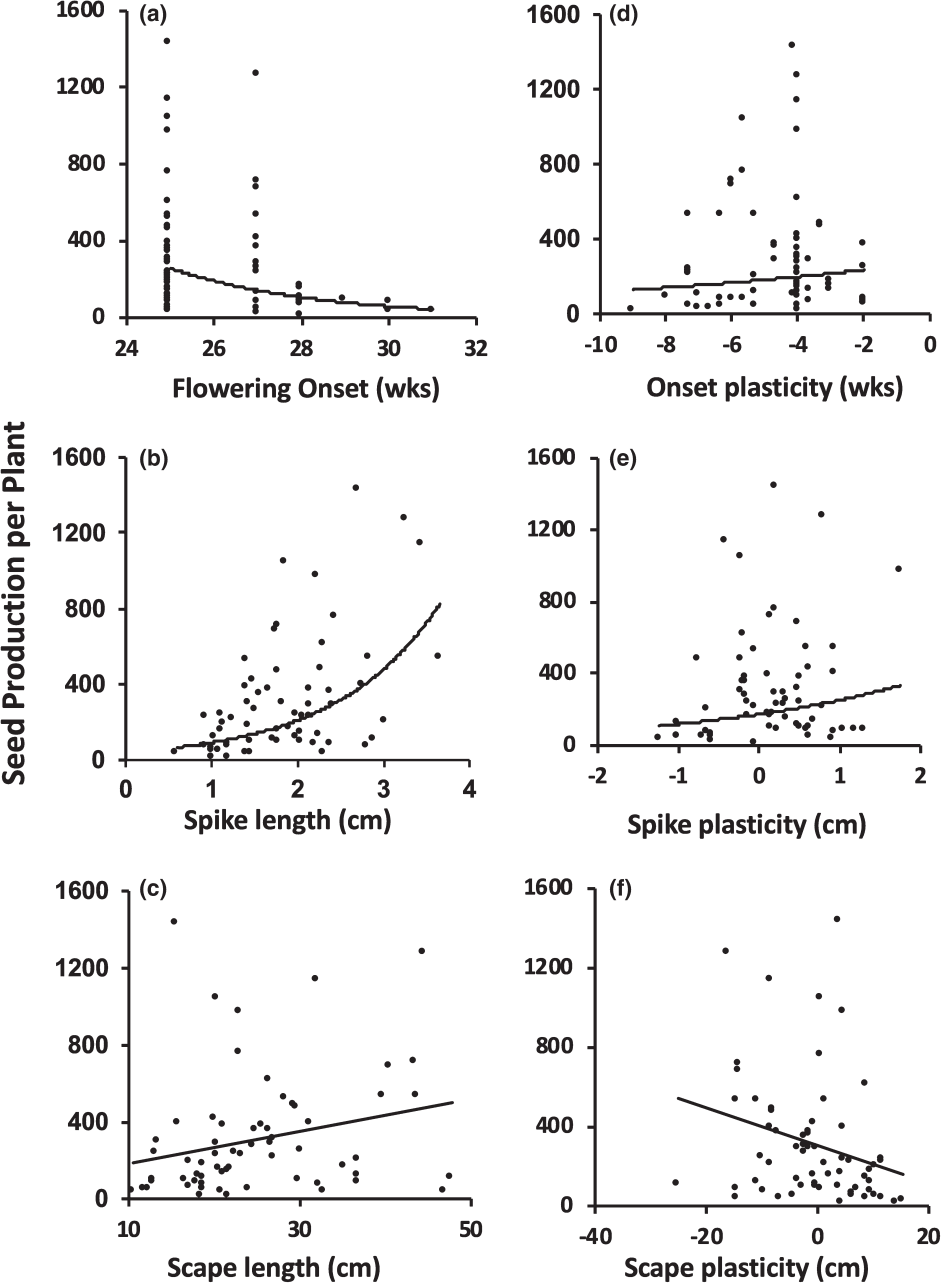
MLBS phenotypic associations between reproductive traits and seed production. Trendlines show quadratic relationships

Fitness showed a downward‐concave relationship with flowering time (Figure 2a; Table 2). Thus, there was evidence for directional selection favoring early flowering, but not too early as indicated by the significant negative quadratic coefficient. In contrast, fitness increased with increased spike length and showed an upward concave relationship with spike length (Figure 2b; Table 2). The significant positive quadratic coefficient (*ɣ_ii_*) indicated that fitness increased more than one would predict under directional selection alone. These combined results provided strong evidence for selection favoring early flowering and long spikes. The curvature for scape length was slightly concave downward, but there was no evidence of selection on this trait (Figure 2c; Table 2A).

**FIGURE 3:**
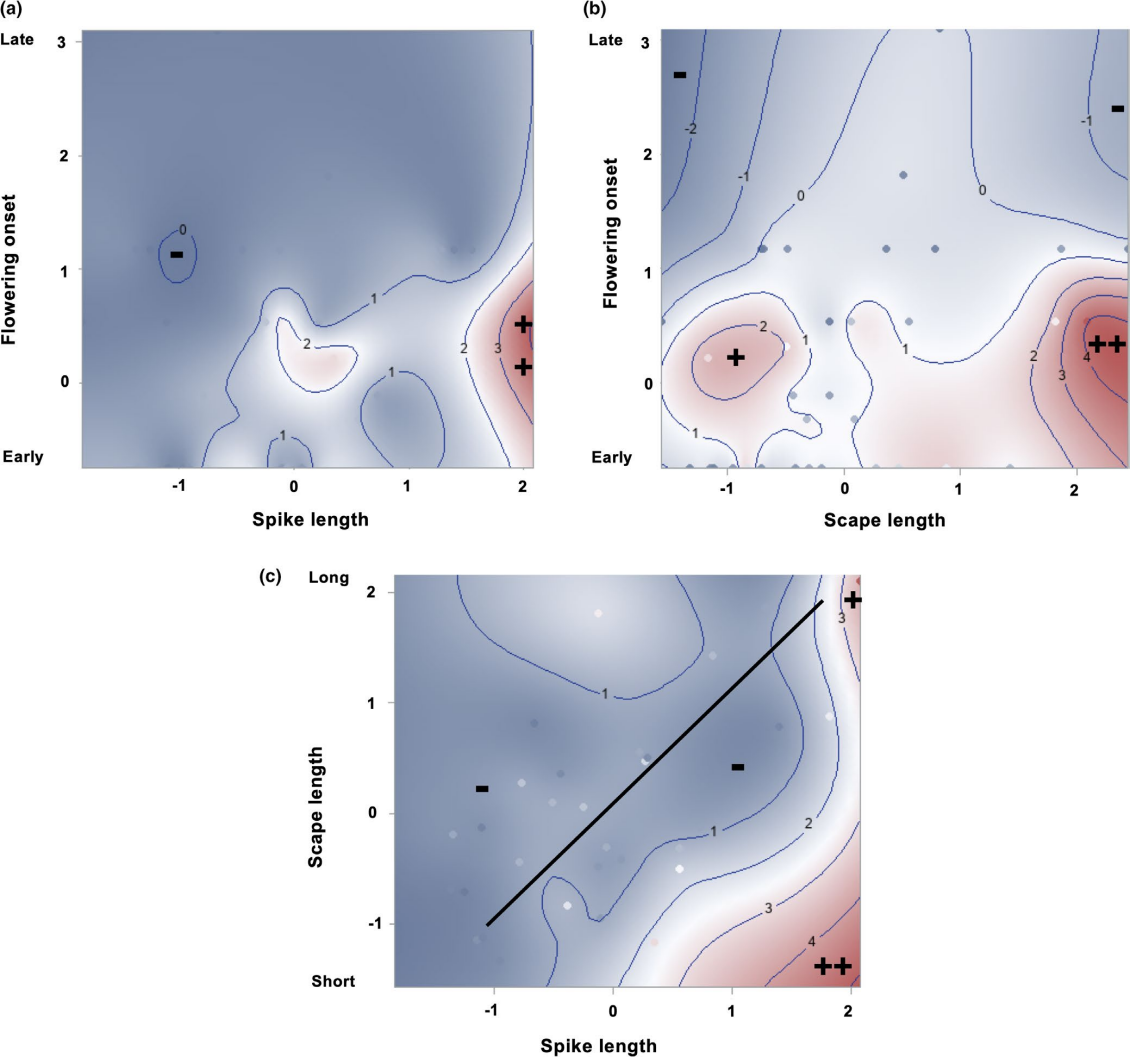
MLBS genotypic fitness surfaces showing correlational selection: (a) flowering onset by spike length, (b) flowering onset by scape length, (c) scape length by spike length. Colors: dark blue = lowest fitness (− sign), dark red = highest fitness (+ or ++ sign). Each contour line represents a line of equal fitness. Values on axes and contour lines show standard deviations (Some genotypes can be seen as dots). Genetic correlation shown by the straight line (*p* < .0001)

All possible cross‐products (*ɣ_ij_*) were statistically significant (Table 2). The cross‐products for onset by spike length and spike length by scape length were negative, whereas, the opposite was true for onset by scape length. These data provided evidence for correlational selection favoring both early‐flowering plants having long spikes with short scapes and late‐flowering plants having short spikes with long scapes. However, the genotypic fitness surfaces (Figure 3a–c) were largely consistent with the former combination, as were both the linear and quadratic selection coefficients. Thus, there was a fitness advantage for the long spike/short scape combination. Interestingly, fitness surfaces and selection results opposed the positive genetic correlation between spike and scape length (Figure 3c).

Patterns of Selection at *NCAT:*

The statistical results for NCAT were more complex than they were for MLBS. A comparison of the full regression model (1) with the “no‐plasticity” model at NCAT showed that the full model (including plasticity) predicted fitness substantially better than the no‐plasticity model (*Χ* ^2^ = 30.1, *p* = .0365, *df* = 18: full −2 log‐likelihood = 111.7; no‐plasticity −2 log‐likelihood = 141.8), This suggested that plasticity contributed substantially to fitness at NCAT.

Comparisons of the reduced models (2–4) with the full model showed that with very little loss of predictive power, we could use “cross‐product” model 3 (only linear and cross‐product predictors) for our selection analysis. The “cross‐product” model did not significantly differ from the full model (*Χ* ^2^ = 4.2 *p* = .6496, *df* = 6, log‐likelihood for cross‐product model = 115.9), and the “cross‐product” model was superior to linear and quadratic models. These latter models showed lower predictive power when compared to the full model, that is, they significantly differed from the full model (difference for linear model *Χ* ^2^ = 35.1, *p* = .0275, *df* = 21, log‐likelihood = 146.8; difference for quadratic model *Χ* ^2^ = 25.7 *p* = .0413, *df* = 15, log‐likelihood = 137.4). Consequently, we used the cross‐product model that included plasticity to examine individual traits at NCAT (Table 2B).

**FIGURE 4:**
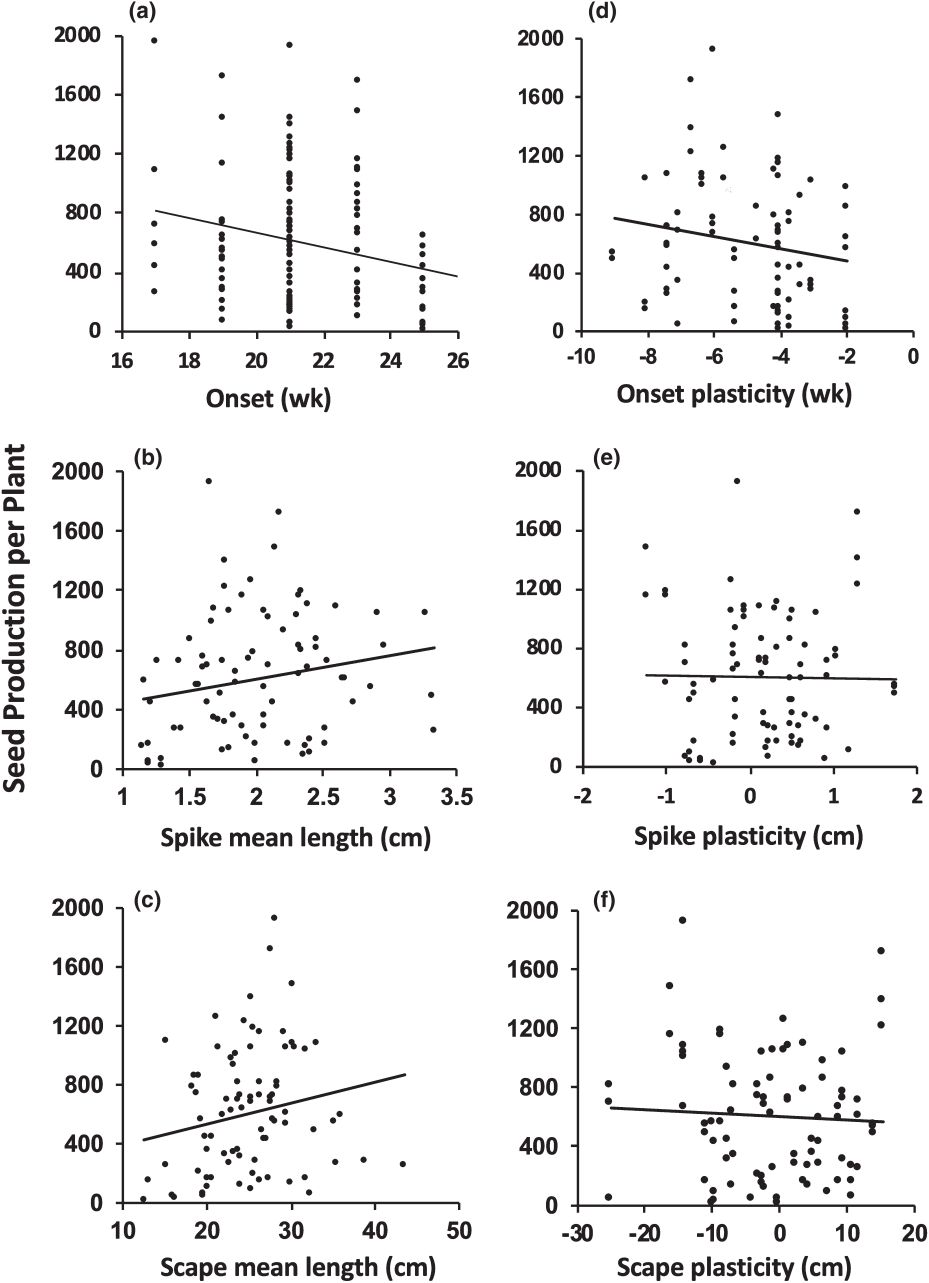
NCAT phenotypic associations between reproductive traits and seed production. Trendlines show quadratic relationships

**FIGURE 5:**
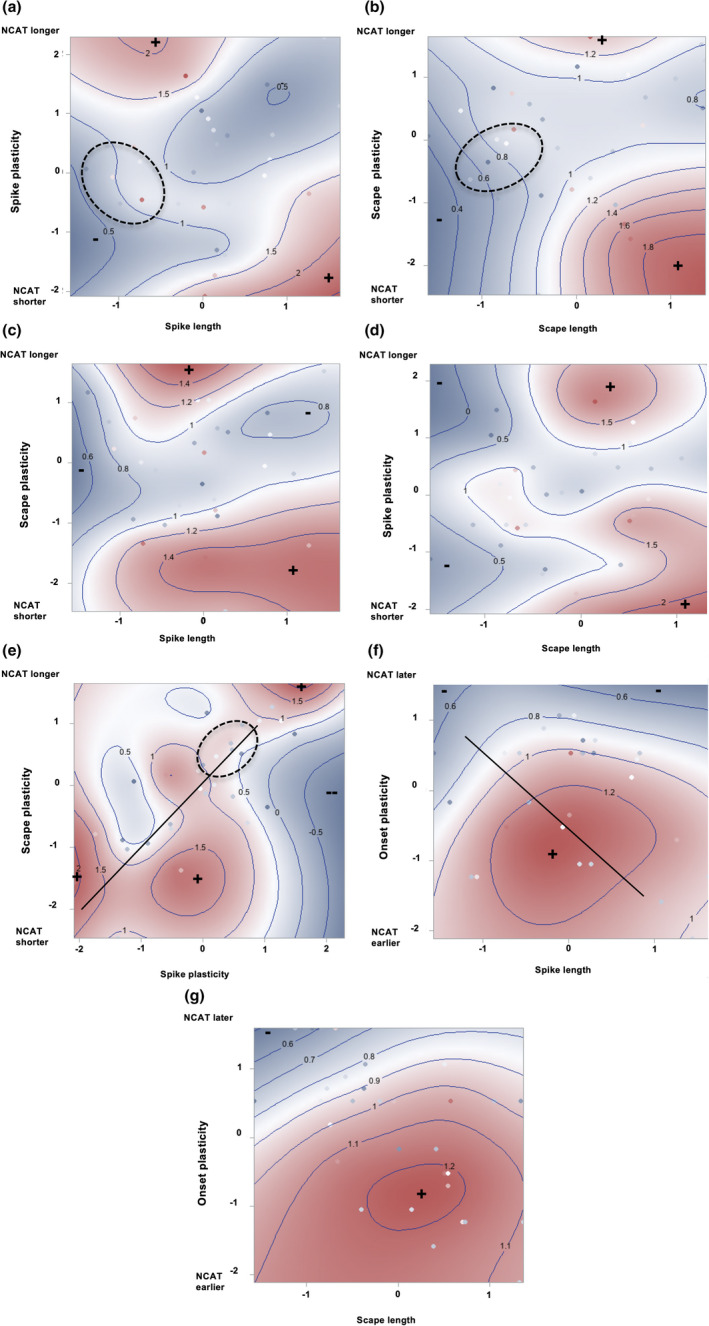
NCAT fitness surfaces showing correlational selection for pairwise combinations of trait values and plasticities: (a) spike length by plasticity, (b) scape length by plasticity, (c) spike length by scape plasticity, (d) scape length by spike plasticity, (e) spike plasticity by scape plasticity, (f) spike length by onset plasticity, (g) scape length by onset plasticity. color:dark blue = lowest fitness (− sign), dark red = highest fitness (+ sign). Each contour line represents a line of equal fitness. Values on axes and contour lines show standard deviations (Some genotypes can be seen as dots). Genetic correlations shown by straight lines (*p* = .0001). The dashed ellipses in (a), (b), and (e) represent three hypothetical samples of six individuals from a source population that have dispersed into a new hypothetical fitness landscape (a, b, or e). Individuals within each ellipse in (a) and (b) have relatively low fitness and the centroid is approximately the same distance from two fitness peaks. Individuals within the ellipse in 5E lie along the saddle of a fitness ridge (See the Discussion for the potential implications)

The regression analysis showed that the intensity and direction of selection for a trait most often depended on the value of another trait, be it a trait value or plasticity. Plasticity's contribution to fitness was manifest only via correlational selection, and all the statistically significant cross‐products involved plasticity of at least one trait (Table 2B). These contributions to fitness were not detected by using linear and quadratic models (Table S1). The quadratic model showed no significant effect of either spike or scape plasticity on fitness (Figure 4e,f). In contrast, the correlational selection model showed disruptive selection on spike and scape plasticity (Figure 5). The only evidence of direct selection in the correlational model was on spike length (Table 2B).

Evidence for correlational selection most frequently involved spike and scape traits. Data for spike length by spike plasticity and scape length by scape plasticity showed evidence of disruptive, negative correlational selection (Figure 5a,b; Table 2B). Each fitness surface showed 2 peaks. One peak showed increased fitness for genotypes producing longer spikes and scapes, as well as “negative” plasticity. Negative plasticity meant that spikes and scapes at NCAT were shorter than at MLBS (genotypic plasticity = NCAT genotypic mean value − MLBS mean value). A second fitness peak was associated with “positive” plasticity (i.e., spikes/scapes at NCAT were longer than at MLBS), coupled with shorter spikes relative to the mean value at NCAT (Figure 5a) and scapes of average length (Figure 5b). These fitness surface patterns were concordant with the negative *ɣ_ij_* values (Table 2).

Fitness surfaces for spike length by scape plasticity and scape length by spike plasticity also each showed two fitness peaks (Figure 5c,d). However, the cross‐products were positive indicating positive correlational selection (Table 2B). For each surface, one fitness peak showed increased fitness for genotypes producing longer spikes and scapes and negative plasticity, as described above. The other peak, however, was shifted toward the mean spike length at NCAT, that is, toward longer spikes (Compare Figure 5a and c) and scapes longer than the mean scape length (Compare Figure 5b and d). Lastly, the fitness surface for spike and scape plasticities (Figure 5e) showed a somewhat wavy ridge extending generally from higher fitness for genotypes producing shorter spikes and scapes at NCAT than at MLBS (lower left) toward higher fitness for those producing longer spikes and scapes at NCAT (upper right). This pattern was consistent with the statistically significant positive *ɣ_ij_* value resulting from the regression analysis (Table 2). The pattern also paralleled the statistically significant genetic correlation between spike and scape plasticities detected at NCAT (Table 1).

Fitness was also influenced by correlational selection involving onset plasticity (Table 2B). The cross‐product for onset plasticity by spike length was negative, which was concordant with the highly significant negative genetic correlation between onset plasticity and spike length (Table 1). In contrast, the cross‐product for onset plasticity by scape length was positive. Fitness surfaces for both cross‐products involving onset plasticity showed only one peak, more a mesa, where fitness was higher for genotypes flowering earlier at NCAT than at MLBS, that is, negative plasticity, over a range of spike and scape lengths close to their mean values (Figure 5f,g).

**FIGURE 6:**
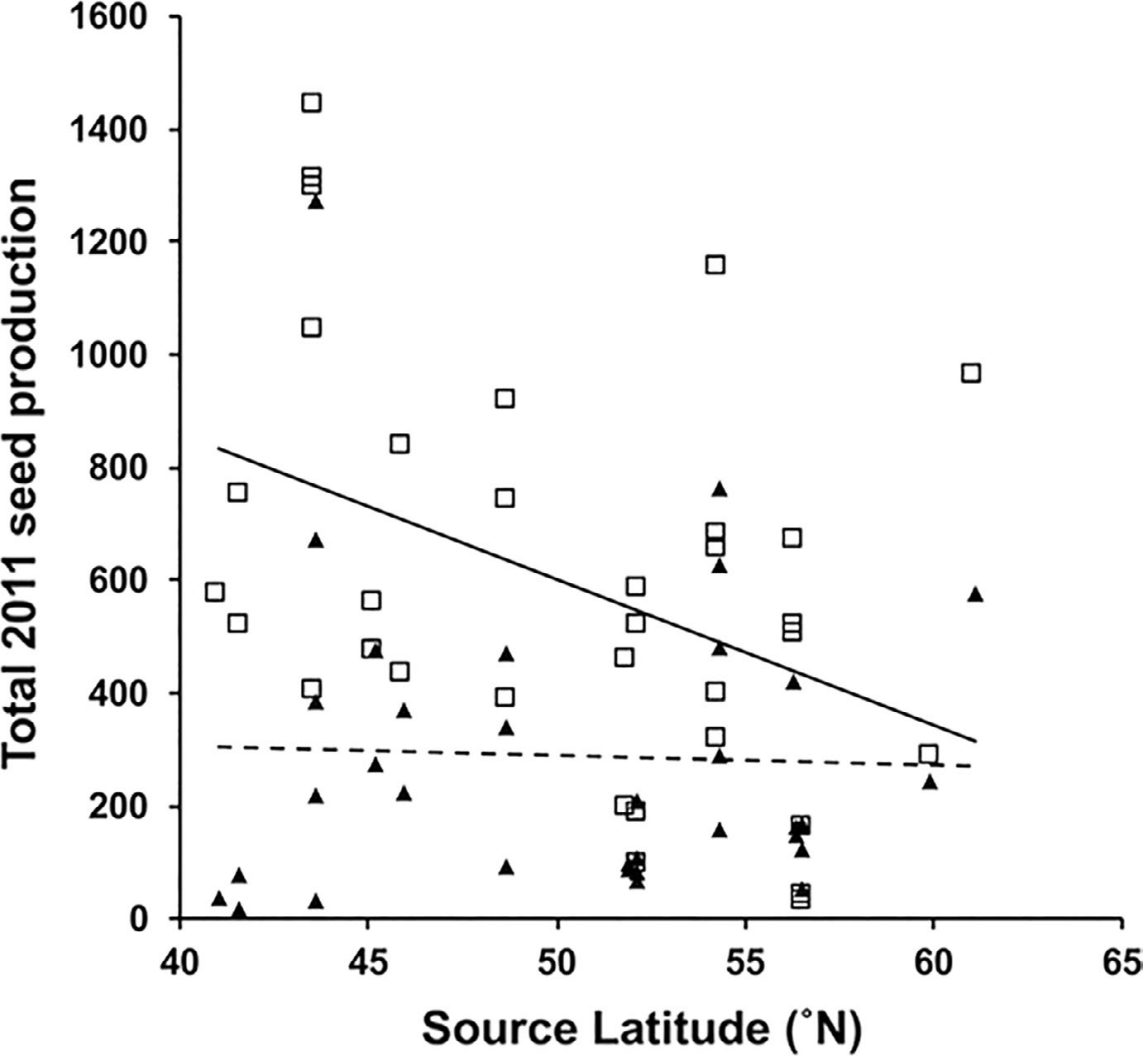
The association between mean seed production per genotype (i.e., average of clones within each genotype) and the source latitude of each genotype shown for transplant sites: MLBS (solid triangles, dashed line) and NCAT (open squares, solid line)

Finally, the analysis showed that parental latitude influenced offspring fitness at NCAT (Figure 6), though not at MLBS (Table 2). Offspring of southern European parents showed higher reproductive output than did offspring of northern parents. This was not unexpected given the warmer temperature regimes during the reproductive season at NCAT (Figure S1), although we did not see the opposite pattern at MLBS.

## 4 DISCUSSION

To our knowledge, this is the first empirical experiment that has estimated linear, quadratic, and correlational selection on trait values and plasticities of a suite of functionally related traits in a natural setting. Our experiment was limited in scope, being restricted to one plot per experimental site and to reproductive traits, and unfortunately, it was not possible to collect data beyond one flowering season. In spite of this, several important aspects of plasticity's role during the early stages of invasion emerge from this study. First, results are consistent with the hypothesis that in a single invasion event, multiple modes of selection can affect a suite of trait phenotypic values and plasticities, that is, genetically based flexibilities that allow for phenotypic modification in response to environmental change. However, which modes of selection are important appear to be dependent on the environment. Second, results show that linear and quadratic selection analyses, alone, can miss the detection of selection on plasticity, as evidenced by comparing the results of the cross‐product model with those of linear and quadratic models. Third, results show how retaining the signs of plasticity can allow one to discriminate between the effects of magnitude and direction of plasticity on fitness and also help one detect correlational selection.

An evolutionary question that has been debated for several decades is whether or not plasticity can be a direct target of selection in nature (e.g., Sarkar & Fuller, 2003; Via et al., 1995). The answer has been elusive both because few studies have simultaneously estimated selection on both a trait value and plasticity in a natural setting and because even fewer have assessed nonlinear, as well and direct, selection (Baythavong & Stanton, 2010; Callahan & Pigliucci, 2002; Donohue et al., 2000; McIntyre & Strauss, 2014; Steinger et al., 2003; Tucić et al., 1998; van Kleunen & Fischer, 2005). Blows and Brooks (2003) noted this problem by showing that the strength of nonlinear selection on sets of multivariate traits has been regularly underestimated as a consequence of ignoring nonlinear correlational selection acting on pairs of traits. While we found no evidence of selection acting on plasticity at MLBS, we observed strong evidence of correlational selection affecting both trait value and trait plasticity with our full model at NCAT. Had we ignored correlational selection in our statistical model, we would have missed this evidence and underestimated plasticity's contribution to the survival and early establishment of our “colonists.”

Targets of selection and evolutionary potential are well known to be environment‐dependent (e.g., Auld et al., 2010; Bégin & Roff, 2001; Brock & Weinig, 2007; Donohue et al., 2001), and our results are consistent with this environmental dependence. In contrast to the NCAT site, we found no evidence that plasticity influenced fitness at the MLBS site. Both of our transplant sites had similar temporal patterns of photoperiod and shade during the reproductive season. However, temperatures differed between transplant sites and changed over the course of the season. For many weedy species that reproduce over several months, abiotic and biotic environments can change as a reproductive season progresses (e.g., for *P. lanceolata*: temperature, seed predation, Lacey et al., 2003). Consequently, selective landscapes are likely to be phenologically dynamic and may help explain why one observes multiple peaks, or ridges, or large mesas on a fitness surface, as we did for spike and scape plasticities. Additionally, nutrient and water availability may have changed phenologically and differed between sites. All have been shown to modify flowering onset and spike and scape lengths (Lacey & Herr, 2005; Primack & Antonovics, 1981; van Tienderen, 1990, 1992), and any could have contributed to the differences in statistical results that we detected between sites.

Several conclusions can be drawn from the NCAT results for *P. lanceolata*. The most obvious is that selection, both direct and correlational, favored longer spikes. From a fitness perspective, this makes intuitive sense. Spike length was strongly correlated with flower number (Herrera, 2013). Longer spikes, that is, inflorescences, increase potential seed production. The second is that correlational selection favored earlier onset of flowering. Given the warmer climate at NCAT, flowering earlier would potentially allow more time for reproduction, which can extend through August if late‐summer rainfall occurs. Also, earlier flowering would allow plants to escape seed predation by grasshoppers, whose populations can explode in mid‐summer in some years (Lacey et al., 2003). Third, fitness surfaces (Figure 5c–e) show that the combinations of long spikes coupled with negative scape plasticity (i.e., shorter scapes at NCAT than at MLBS) or with no plasticity in scape length were less fit than the combination of long spikes and positive plasticity (i.e., longer scapes at NCAT than at MLBS). This result is consistent with the hypothesis that this trait–plasticity combination is advantageous in warmer climates. Longer scapes raise spikes farther from the ground, likely facilitating the cooling of reproductive tissues. Likewise, scape shortening in cooler climates, for example, MLBS, can increase the potential for both radiative warming from the ground and protection from cold winds. Future research could further test this hypothesis.

Because natural selection acts on multiple traits simultaneously in nature, many evolutionary biologists have argued that correlational selection should be common, especially for functionally related traits (Blows, 2007; Blows & Brooks, 2003; Brodie & McGlothlin, 2007; Lande & Arnold, 1983; Phillips & Arnold, 1989; Schluter & Nychka, 1994). Moreover, correlational selection is posited to be a strong evolutionary force by which traits become functionally and genetically integrated (Brodie, 1992; Lande, 1980, 1984). Few empirical measures of correlational selection on a suite of functional traits in nature have existed to test this belief (Blows & Brooks, 2003; Brodie et al., 1995; Kingsolver et al., 2001; Nicolaus et al., 2016; Sinervo & Svensson, 2002; Wise & Rausher, 2016), and we have found only one plant study that provides evidence of correlational selection on plasticity: shade‐dependent leaf‐length plasticity in *Iris* (Tucić et al., 1998). For this reason, our study is noteworthy. We detected evidence for correlational selection acting on key reproductive traits, values, and plasticities, where *P. lanceolata* grows naturally. Results are consistent with the above argument that correlational selection can influence evolutionary change in a population and population responses to novel habitats. Had our data set been large enough to include 3‐way interactions in a more complicated regression model, we might have detected more peaks and valleys on our fitness surface. However, there is still a need to develop analytical techniques that can integrate information from more complex models in a biologically understandable way (Brodie & McGlothlin, 2007; Brodie et al., 1995).

A colonizing species enters a new habitat with a set of genetic correlations among functionally related traits, and these correlations could constrain or facilitate adaptation to the new habitat. Our data showed evidence of both possibilities. At MLBS, correlational selection favored genotypes with long spikes and short scapes (Figure 3c). However, the positive genetic correlation between spike and scape length of our European “colonist” experimental population would be likely to constrain evolutionary change in that direction. In contrast, at NCAT, spike and scape plasticities were positively genetically correlated with each other, perhaps reflecting the single developmental pathway controlling growth in these structures. At this site, however, the fitness surface produced from these data suggested a ridge of higher fitness rather than a peak (Figure 5e), and the ridge loosely paralleled the direction of the genetic correlation. This ridge could allow evolutionary change toward increased fitness in more than one direction. In general, correlational selection should promote and maintain genetic correlations (Cheverud, 1984; Lande, 1980, 1984), If so, we would predict that this genetic correlation between spike and scape plasticities would be strengthened.

Our data suggest that correlational selection can influence the direction of early evolutionary change, depending on where dispersing individuals land on an adaptive landscape. While the true adaptive landscape in the North Carolina Piedmont, where the NCAT plot was located, is not known, our fitness surfaces suggest that multiple peaks, ridges, or even mesas of higher fitness could characterize a landscape for an introduced species. Such topographical variation creates the potential for multiple responses to correlational selection. For example, suppose that a few individuals arriving from a source population (e.g., those within the dashed ellipses in Figure 5a,b) are deposited over multiple years in a fitness valley (e.g., Figure 5a,b). Such situations would have been common in the 16th and 17th centuries when multiple commercial ships arrived in North America and rid themselves of ballast to make room for goods to be carried back to Europe. While the individuals in 1 year might establish and evolve toward one fitness peak, in a later year, other individuals from the same population might evolve toward a different fitness peak. In this way, multiple fitness peaks arising from negative disruptive correlational selection at environmentally similar locations might facilitate multiple divergences from the source population, leading to incipient speciation in a new region. Alternatively, if individuals (e.g., those within the dashed ellipse in Figure 5e) were to land on a fitness ridge, as in Figure 5e, correlational selection could facilitate expansion along the ridge, maintaining genetic diversity. While these are hypothetical scenarios, they illustrate the value of experiments that can measure correlational selection.

Our experiment differed in two methodological ways from those that have previously explored selection on plasticity in nature. First, rather than using trait value means averaged across transplant sites for our analyses, we estimated the effects of plasticities against trait values expressed in a single environment. This allowed us to assess the independent effects of a trait value and its plasticity in each environment where selection occurred. Second, we retained the signs of plasticity values in statistical analyses to explore fitness effects. This allowed us to examine both the magnitude and direction of plasticity on fitness.

Biologists estimating plasticity's adaptive significance in nature have often used absolute values of plasticity to assess its costs and benefits (e.g., Palacio‐Lopez et al., 2015; van Kleunen & Fischer, 2005, 2007; Volis, 2009; Wang et al., 2018). However, this has likely obscured the fitness effects of plasticity (e.g., Auld et al., 2010; de Witt et al., 1998; Murren et al., 2015; Scheiner & Berrigan, 1998; van Buskirk & Steiner, 2009; van Kleunen & Fisher, 2005, 2007; Weinig et al., 2006). Relyea (2002), van Kleunen and Fischer (2005, 2007), Weinig et al. (2006), and Dechaine et al. (2007) pointed out that using absolute values can confound the magnitude and direction of plasticity, potentially obscuring fitness effects, and our results illustrate this point in the context of correlational selection. For example, if one visually folds Figure 5a or 5b along the line of zero plasticity to produce a new 3D fitness surface that ignores direction (i.e., used absolute values), the two distinct fitness peaks disappear. Had we not retained the signs in our analysis, we could not have detected the fitness benefit of producing longer spikes and scapes at NCAT than at MLBS. Negative disruptive correlational selection for these plasticities would not have been detected. In another example, we could not have observed that earlier flowering at NCAT relative to MLBS, as opposed to plasticity in the opposite direction, was favored at NCAT (Figure 5e,f).

Auld et al. (2010) pointed out that estimates of selection coefficients, and thus costs and benefits of plasticity, arising from multivariate regression analyses could be biased when two predictors are highly correlated, but only one is correlated with fitness. Because of this problem, Auld et al. urged that data from relevant published studies be reanalyzed. We detected only one such case in the NCAT data. Flowering onset and plasticity were highly genetically correlated but only onset plasticity was found to affect fitness. However, we detected this fitness effect by including direction as well as magnitude of plasticity in our analyses and by including correlational selection in our regression model (Figure 5f). Thus, it is unclear how much, or if, there was identifiable bias. Correlational selection and signs for plasticity have typically not been included in studies examining costs of plasticity (See references cited above). Therefore, in addition to the suggestion by Auld et al. (2010) to reanalyze data from published studies in order to examine the genetic correlations between trait values and plasticities, we urge that data be reanalyzed to include the direction of plasticity and correlational selection in regression models.

In conclusion, our study illustrates the importance of examining all modes of selection when assessing the fitness effects of plasticity when a species experiences habitat change, not only during invasions, but also more generally during habitat modification, for example, via urbanization, climate change. Also, the study reinforces the call for retaining the signs of plasticity in statistical analyses. Future studies might consider addressing these questions: (a) What is the strength of correlational, in addition to linear and quadratic selection, on suites of functionally related trait values and plasticities; (b) what are the fitness effects of plasticity in a suite of traits, when we account for direction and magnitude, that is, the signs, of plasticity; (c) what are the genetic correlations among trait values and plasticities in a group of individuals arriving in a novel habitat? (d) How might these correlations influence the group's responses to linear and nonlinear selection? Addressing these questions, we believe could provide new insights about plasticity's roles during early invasions, and perhaps also, in responding generally to habitat change.

## CONFLICT OF INTEREST

The authors disclose no conflicts.

## AUTHOR CONTRIBUTIONS

**Elizabeth P. Lacey:** Conceptualization (lead); Data curation (supporting); Formal analysis (lead); Funding acquisition (lead); Investigation (supporting); Methodology (lead); Project administration (lead); Resources (lead); Software (equal); Supervision (lead); Validation (lead); Visualization (lead); Writing‐original draft (supporting); Writing‐review & editing (lead). **Freddy O. Herrera:** Conceptualization (supporting); Data curation (equal); Formal analysis (supporting); Funding acquisition (supporting); Investigation (equal); Methodology (equal); Project administration (supporting); Resources (supporting); Software (supporting); Supervision (supporting); Validation (supporting); Visualization (supporting); Writing‐original draft (lead); Writing‐review & editing (supporting). **Scott J. Richter:** Conceptualization (supporting); Data curation (supporting); Formal analysis (equal); Funding acquisition (supporting); Investigation (supporting); Methodology (supporting); Resources (supporting); Software (equal); Supervision (supporting); Validation (equal); Visualization (equal); Writing‐original draft (supporting); Writing‐review & editing (equal).

## ETHICAL APPROVAL

This manuscript is not published elsewhere, and we have used no animals in this study.

## Supporting information

Figure S1

Figure S2

Figure S3

Figure S4

Table S1

## Data Availability

The data are available on Dryad (https://doi.org/10.5061/dryad.wwpzgmsj1).
