# Supplementary figures and images for: Multiple modes of selection can influence the role of phenotypic plasticity in species' invasions: Evidence from a manipulative field experiment
Source: Ecol Evol. 2021 Mar 9;11(9):4140–57. doi: 10.1002/ece3.7311 (PMC8093752; doi:10.1002/ece3.7311)

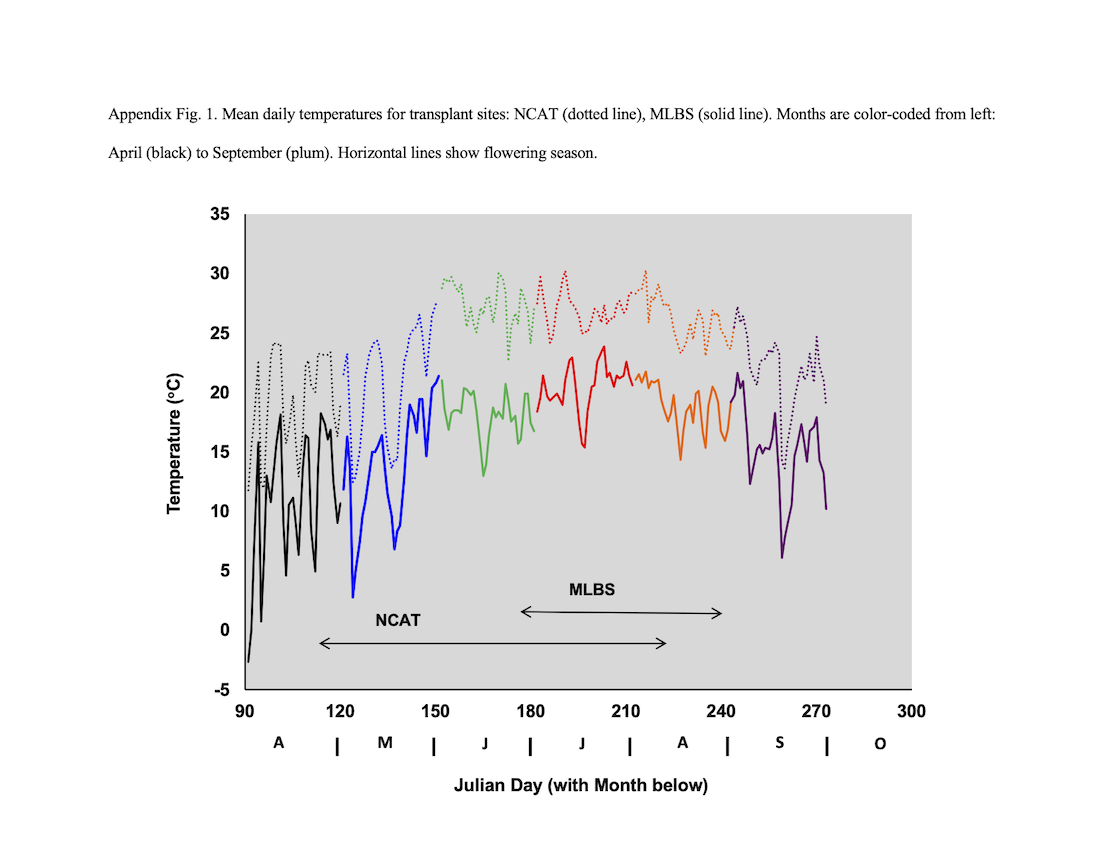

Supplement: Supplementary file 1 — Figure S1 [file ECE3-11-4140-s005.tiff]

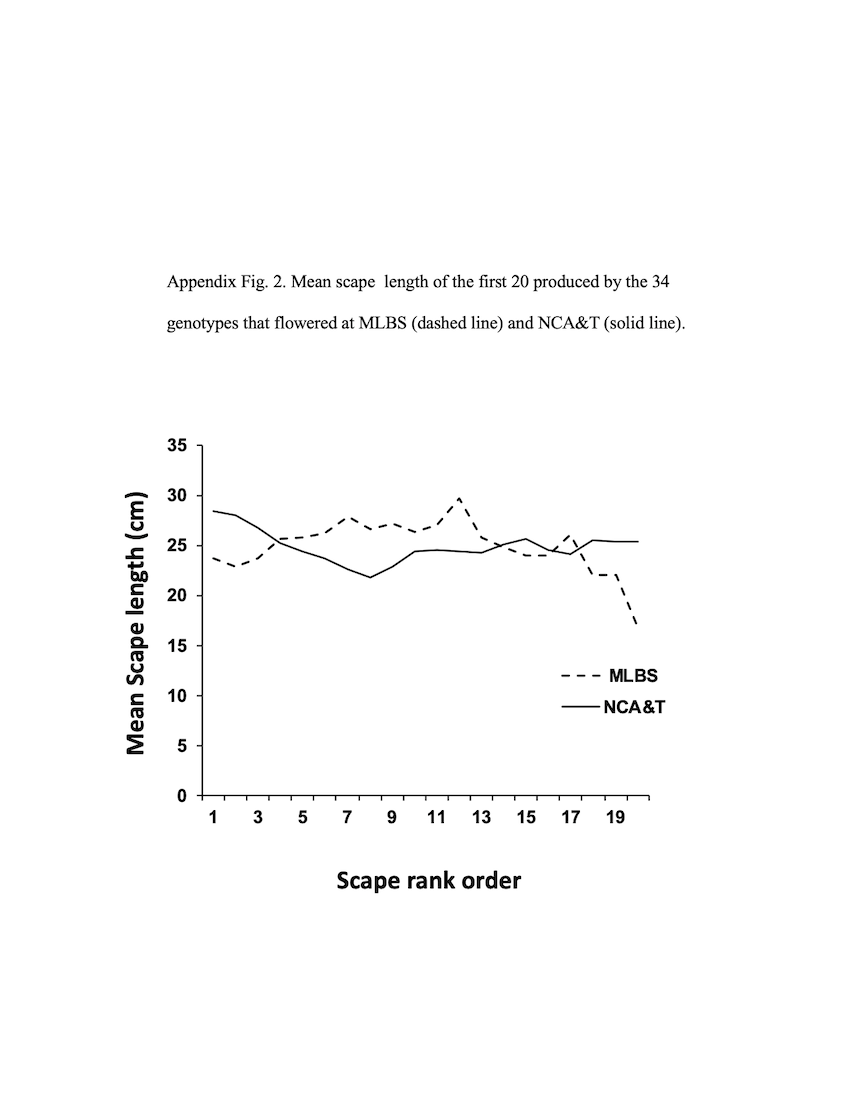

Supplement: Supplementary file 2 — Figure S2 [file ECE3-11-4140-s001.tiff]

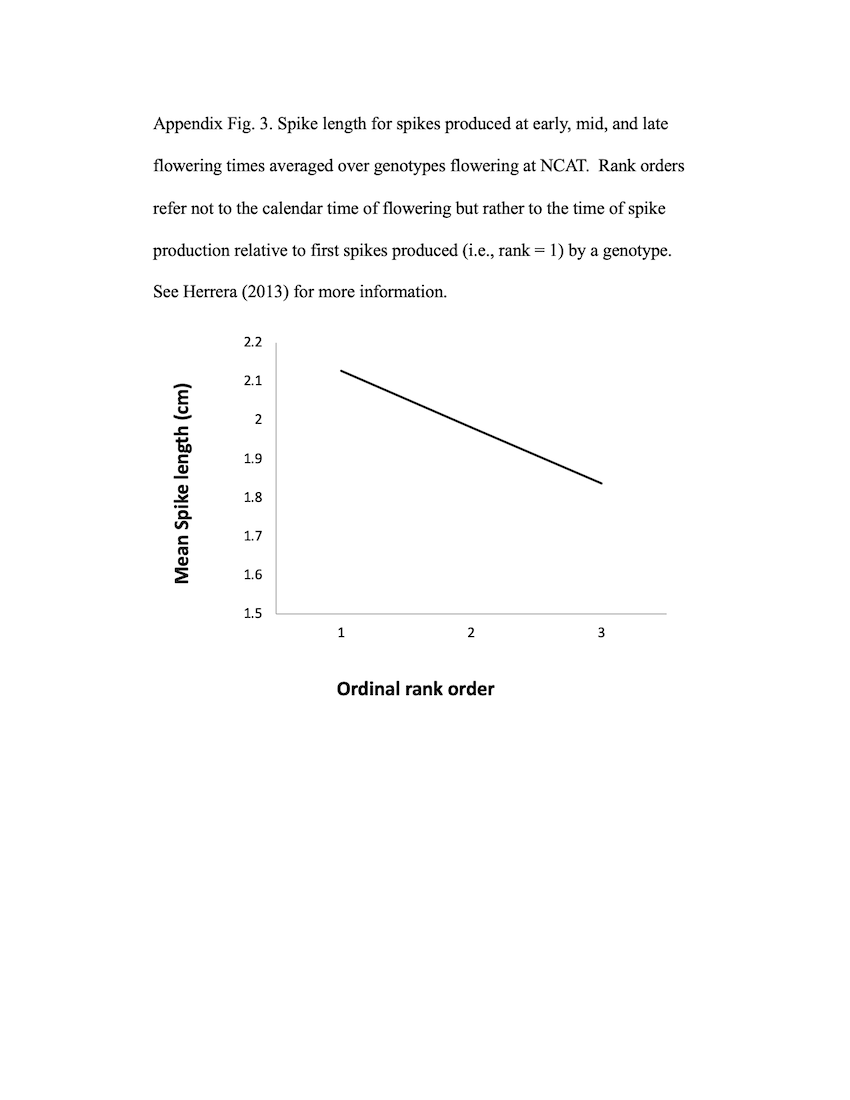

Supplement: Supplementary file 3 — Figure S3 [file ECE3-11-4140-s004.tiff]

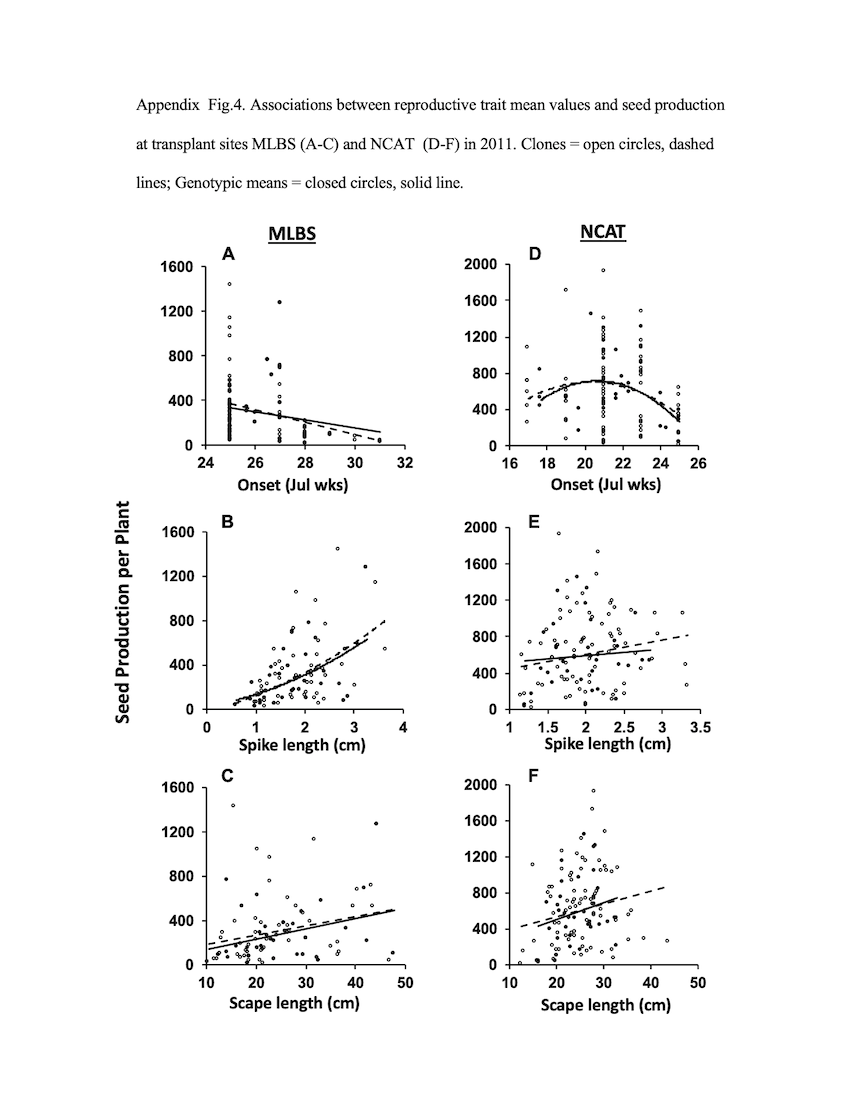

Supplement: Supplementary file 4 — Figure S4 [file ECE3-11-4140-s003.tiff]
