# Supplementary material for: Multiple modes of selection can influence the role of phenotypic plasticity in species' invasions: Evidence from a manipulative field experiment
Source: Ecol Evol. 2021 Mar 9;11(9):4140–57. doi: 10.1002/ece3.7311 (PMC8093752; doi:10.1002/ece3.7311)
Supplement: Supplementary file 5 — Table S1 [file ECE3-11-4140-s002.docx]

Appendix Table 1. Linear and quadratic selection coefficients (± 1 SE) and p values from NCAT phenotypic selection analysis for effects of trait values and plasticities on fitness. Traits = flowering onset time, spike length, and scape length. All quadratic values are doubled. Linear coefficients are from the linear model.

| **Variable** | **Linear (β)** | | | **P** | **Quadratic (2γ_ii_)** | | | **P** | | |  |  |  |
| --- | --- | --- | --- | --- | --- | --- | --- | --- | --- | --- | --- | --- | --- |
| Onset time | -0.2016 ± 0.1655 | | | 0.2294 | -0.0008 ± 0.2580 | | | 0.9974 |  | | | |  |
| Onset plasticity | 0.0391 ± 0.1282 | | | 0.7627 | -0.0546 ± 0.1030 | | | 0.6005 |  | | | |  |
| Spike length | 0.2408 ± 0.1078 | | | 0.0304 | -0.1332 ± 0.1504 | | | 0.3809 |  | | | |  |
| Spike plasticity | -0.2385 ± 0.1766 | | | 0.1873 | 0. 0106 ± 0.1652 | | | 0.9497 |  | | | |  |
| Scape length | -0.0110 ± 0.1192 | | | 0.9268 | -0.2776 ± 0.1426 | | | 0.0581 |  | | | |  |
| Scape plasticity | 0.0951± 0.1755 | | | 0.5922 | 0.1026 ± 0.1720 | | | 0.5563 |  | | | |  |
| Latitude | -0.0461 ± 0.0211 |  | 0.0189 | | |  |  | | |  | |  |  |
